# Subjectively different but objectively the same? Three profiles of QoL in people with severe mental health problems

**DOI:** 10.1007/s11136-018-1964-7

**Published:** 2018-08-13

**Authors:** David C. Buitenweg, Ilja L. Bongers, Dike van de Mheen, Hans A. M. van Oers, Chijs van Nieuwenhuizen

**Affiliations:** 1 Scientific Center for Care and Welfare (Tranzo), Tilburg University, PO BOX 90153, 5000 LE Tilburg, The Netherlands; 2 GGzE Institute for Mental Health Care, Eindhoven, The Netherlands; 3 IVO Addiction Research Institute, Erasmus Medical Center, Rotterdam, The Netherlands; 4 National Institute for Public Health and the Environment, Bilthoven, The Netherlands

**Keywords:** Quality of life, People with severe mental health problems, Latent class analysis

## Abstract

**Purpose:**

Quality of life (QoL) is a broad outcome that is often used to assess the impact of treatment and care interventions in mental health services. QoL, however, is known to be influenced by individual values and preferences. To investigate this heterogeneity on the individual level, this study aimed to distinguish classes with distinct QoL profiles in a broad group of people with severe mental health problems and to identify the QoL domains that are most strongly related to the classes.

**Methods:**

QoL data of seven studies that used the Lancashire quality of life profile (LQoLP) were used in a latent class analysis. Sociodemographic variables, health-related variables, and measures of well-being were used to characterise the classes. Additionally, univariate entropy scores were used to assess the strength of the association between the ten LQoLP domains and the latent classes.

**Results:**

Two of the three indices of fit pointed towards a three-class model. The three classes differed significantly on all of the LQoLP domains, on well-being, and on ‘being in an intimate relationship’. No differences were found for the majority of the health-related and sociodemographic variables. The LQoLP domains ‘family relations’, ‘positive self-esteem’, and ‘negative self-esteem’ were most strongly related to the latent classes.

**Conclusions:**

The identification of three distinct classes of QoL scores re-emphasises the heterogenic nature of QoL. The lack of differences in sociodemographic or health-related characteristics between the three classes suggests that QoL is primarily determined by subjective, personal evaluations, rather than by objective characteristics and circumstances.

## Introduction

Since the 1980s, quality of life (QoL) has become increasingly important as a patient-reported outcome (PRO) in mental health services [1–4]. In mental health, QoL is defined as an individuals’ subjective evaluation of various life domains, such as physical health, family relations, finances, and well-being [5, 6]. Scores on these domains are often combined to form a global QoL score [4]. Due to its broad scope, QoL assessment in mental healthcare is useful for evaluating the impact of treatment and care interventions [7, 8]. The use of QoL data in mental health may even improve patients’ satisfaction with care [9, 10]. As a consequence, QoL is widely regarded as an important, if not essential, outcome measure for people with mental health problems [9, 11, 12]. The broadness of QoL is one of its main strengths, but it also introduces complexity and results in a multitude of scores on the domain and global level [13]. The strong subjective aspect of QoL enhances this complexity. The concept is known to be influenced by individual priorities and values and differs between individuals [14] and even—because of response shift—within individuals [15–17].

To improve our understanding of the QoL of people with mental health problems, and to facilitate the interpretation of QoL scores, many researchers have investigated the relationships between QoL and demographic, clinical, and personal variables, such as age [18], country of residence, employment, accommodation [19], frequency of contact with family [20], severity of symptoms [20–22], insight [21], coping [18, 21], and medication adherence [18]. While these studies have advanced our understanding of the factors influencing QoL in mental health, such studies disregard potential heterogeneity on the individual level as they are focusing on average group scores.

The importance of the heterogeneity of QoL has been underlined in recent research [21, 23, 24]. Three studies illustrate how QoL differs within groups as a function of individual characteristics. Priebe and colleagues [23], for instance, identified a significant association between employment and QoL. This association was stronger for patients with neurotic disorders compared to patients with mood disorders or schizophrenia. A similar difference was found for the association between symptom levels and QoL [23]. A study by Montemagni and colleagues [21] provides a second example. The researchers investigated the associations between QoL and negative symptoms, insight, and coping strategies in a group of outpatients with schizophrenia. Their results indicate that correct attribution of symptoms to illness positively influences QoL in patients with mild negative symptoms, but not in patients with severe negative symptoms [21]. In an attempt to gain more insight into the heterogeneity in QoL scores, De Maeyer and colleagues [24] used latent class analysis (LCA) to explore classes with distinct QoL profiles within a homogeneous sample of opiate-dependent individuals. The three classes identified using LCA were characterised using sociodemographic, drug-, health-, and person-related variables. The first class consisted of individuals living in marginal conditions who had problems regarding housing, judicial problems, and frequently demonstrated injected behaviour. Another class involved socially included opiate-dependent individuals whom experienced problems with severe mental health problems, goal fulfilment, and employment. Hence, the identification of classes with distinct QoL profiles may be beneficial to the ability to interpret and apply QoL data in an individualised way.

The aim of this study is to investigate classes with distinct QoL profiles in a broad group of people with severe mental health problems. Furthermore, to facilitate the interpretation of QoL scores, the QoL domains that are most strongly related to the classes will be identified.

## Materials and methods

### Sample

This study involved a secondary analysis of QoL data collected with the Dutch version of the Lancashire quality of life profile (LQoLP). The LQoLP is a structured interview specifically developed to assess the QoL of people with severe mental health problems [25, 26]. To identify relevant data sets, a number of colleagues were consulted by telephone and email. Inclusion criteria were that the data sets targeted people with severe mental health problems and used the original Dutch version of the LQoLP [4] or the extended Dutch version of the LQoLP [26]. Data sets fitting these criteria were collected and combined into a single database.

Seven data sets were included [5, 24, 26–30]. In the case of a longitudinal design, only the measurement at the first time point was used. LQoLP data for 1277 persons with psychiatric problems were available. The data sets were collected between 1997 and 2014. Table 1 provides an overview of the characteristics of the seven included studies.

**Table 1:** Study characteristics of the seven included studies

Study	Sample size	Research design	LQoLP version
Proost [30]	116	Cross-sectional	Original
Van Nieuwenhuizen et al. [26]	487	Cross-sectional	Original
Barendregt et al. [27]	172	Longitudinal	Extended
De Maeyer et al. [24]	159	Cross-sectional	Extended
Bouman et al. [28]	135	Cross-sectional	Extended
Harder et al. [29]	164	Longitudinal	Extended
Van Nieuwenhuizen and Nijman [5]	44	Cross-sectional	Extended

### Lancashire quality of life profile

The LQoLP measures an individuals’ satisfaction with ten different life domains, as well as their general well-being. The LQoLP contains both objective items (‘Do you have a paid job?’) and subjective items (‘How satisfied are you with your monthly income?’). The LQoLP generates a QoL profile that is based on 58 subjective items. Objective items are included in the interview because variance in global well-being has been found to be mediated by both objective and subjective well-being [25] and to serve as a primer.

All of the ten LQoLP domains comprising the subjective QoL profile were used in the analysis: (1) ‘physical and mental health’, (2) ‘leisure and social participation’, (3) ‘finances’, (4) ‘safety’, (5) ‘living situation’, (6) ‘family relations’, (7) ‘positive self-esteem’, (8) ‘negative self-esteem’ (Domain 7 and Domain 8 were measured using a modified version of the Self-Esteem Scale [31]), (9) ‘framework’, and (10) ‘fulfilment’ (Domain 9 and Domain 10 were measured by the Life Regard Index [32]). Both the Self-Esteem Scale and the Life Regard Index are part of the LQoLP [26]. Domain scores were calculated by averaging item scores.

The first six domains cover tangible aspects of QoL and are measured on a 7-point Likert scale, ranging from ‘cannot be worse’ (1) to ‘cannot be better’ (7). The last four domains involve intangible, self-related aspects of QoL and are measured on a 3-point Likert scale: ‘disagree’ (1), ‘I do not know’ (2), and ‘agree’ (3). To allow comparison between all domains, scores on the last four domains were transformed using the following transformation *M′* (transformed mean score) = [*M* (mean score)/3] × 7 [4]. A QoL score of below 4 has been defined as a low QoL score and a QoL score of 4 or higher has been designated as a high QoL [5]. The LQoLP also contains two measures of global well-being in the form of Cantril’s Ladder [33] and an average life satisfaction score (LSS; ‘how satisfied are you with life as a whole?’). Additionally, the LQoLP includes a Happiness Scale that asks respondents to report how happy their life has generally been on a 5-point Likert scale. Several variables of the LQoLP, including sociodemographic variables, health-related variables, and measures of well-being were used to characterise the classes. For an overview of these variables, see Table 4.

Psychometric properties (internal consistency, reliability, and validity) of both the original LQoLP and its (extended) Dutch version have been demonstrated to be satisfactory [4, 25, 26]. The Cronbach’s alpha for the 58-item QoL score was 0.93 and eight of the ten domains had an alpha of more than 0.70 [26]. The Intraclass Correlation Coefficients (ICC) for the 58-item QoL score was 0.92, while seven of the ten domains had an ICC of > 0.80. The content validity was guaranteed through the construction process and the construct validity was examined by computing correlations between the 58-item QoL score and the Satisfaction with Life Scale (*r* = 0.71) and a single-item Life Satisfaction Scale (*r* = 0.73) [26].

### Missing data

Due to differences between the original and extended versions of the Dutch LQoLP, three of the ten domains contained missing data. Specifically, two types of missing data were encountered and dealt with using two different methods. First, in the extended version of the Dutch LQoLP, two out of six items in the domain ‘living situation’ were dropped because they applied to less than 25 percent of the respondents [26]. Consequently, all of the data for the extended Dutch LQoLP were missing on these two items. Due to the large number of cases with missing data on these items, domain scores for all participants were computed based on the four remaining items in the extended Dutch LQoLP. Second, in the extended Dutch version of the LQoLP, items were added to the domain ‘family relations’ (four items) and the domain ‘safety’ (three items), because of the relatively low reliability of these two domains in the original version [26]. Consequently, all data for the original LQoLP version contained missing data on these newly added items. Because missing items were explained by the difference in LQoLP versions, full information maximum likelihood (FIML) was used to address missing data. FIML estimates a likelihood function for every individual, based on the data available for that individual. Model fit information is derived by summing these individual likelihood functions. FIML has been found to be a reliable method when missing data are missing at random (MAR) [34, 35].

### Statistical analysis

To identify classes with distinct QoL profiles based on the patterns of scores on the ten LQoLP domains, an LCA was performed. In LCA, the modelled latent variable is assumed to be categorical, consisting of multiple classes. Individuals are assigned to one of the classes by examining the underlying structure of categorical data [36, 37]. The current analysis consisted of three steps. In the first step of the analysis, LCA models with a varying number of classes were estimated and compared. The analysis started by estimating a model with a single class. Next, models with k + 1 classes were estimated, up to k = 6 classes. These models were compared using three indices of model fit: the Bayesian Information Criterion (BIC), the Vuong–Lo–Mendell–Rubin (VLMR) likelihood ratio test, and entropy. The BIC is an indicator of relative model fit. Lower values indicate a better fit of the model to the data. The VLMR likelihood ratio test compares the relative fit of a model with k classes and a model with k − 1 classes. A significant result on the VLMR test result indicates a better fit of the model with k − 1 classes. Entropy is a measure for the distinctiveness of the classes. Values range from 0 to 1 and a value of 0.8 or higher is generally considered desirable as it indicates a clear delineation of the classes [38]. Model selection depended on these three indices of fit, as well as a theoretical interpretation of the classes. Additionally, univariate entropy [39] was used to assess the contribution of the ten LQoLP domains to the classification. Univariate entropy is a measure of how well the latent indicators identify the latent classes.

In the second step of the analysis, individuals were assigned to one of the classes on the basis of posterior class membership probabilities. The third step of the analysis involved the characterisation of the classes by relating class membership to (1) sociodemographic variables, (2) health-related variables, and (3) measures of well-being. Differences between the classes were investigated using Chi-square tests (for dichotomous variables) or a one-way Analysis of Variance (ANOVA). For variables that violated the assumptions of ANOVA, a non-parametric alternative in the form of a Kruskal–Wallis Test [40] was used. The LCA was performed using M-plus 7.3 [41]. All other analyses were run using SPSS, version 19 [42].

## Results

### Sample characteristics

Participants were predominantly male (72%), with a mean age of 35.16 years (SD = 15.01, range = 12–85). The majority (81.9%) of participants were of Dutch nationality, 16.4 percent of the respondents were employed, and about a third (29.8%) were in an intimate relationship at the time of the interview.

### Latent class analysis

**Fig. 1:**
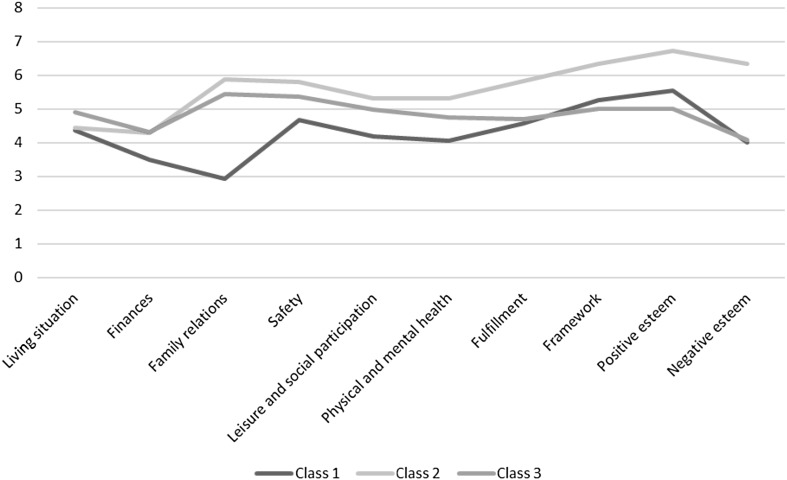
Mean LQoLP domain scores for the three classes identified with the LCA

Fit statistics for latent class models with 1–6 classes are presented in Table 2. Bayesian information criterion (BIC) values decreased across the tested models, which suggested that the 6-class model provided the best fit. The results for the Vuong–Lo–Mendell–Rubin (VLMR) likelihood ratio test, however, revealed that models with more than three classes overfit the data because the test returned a non-significant result for these models (*p* value ≥ 0.05). The three-class model had both a lower BIC score (BIC = 64303.46) and a higher entropy (0.86) than the two-class model (BIC = 64515.92, entropy = 0.83). Although the four-class model had the most favourable entropy (0.9), it also produced a non-significant result on the VLMR likelihood ratio test and contained a relatively small fourth class. Therefore, the three-class model fits the data best. Average QoL scores on the ten LQoLP domains differed significantly between the three classes and can be found in Fig. 1 and Table 3.

**Table 2:** Fit statistics for latent class models with 1–6 classes (*N* = 1277)

Number of classes	BIC	Entropy	Vuong–Lo–Mendell–Rubin test *p* value
1	68,016.76		
2	64,515.92	0.83	0.00
3	64,303.46	0.86	0.013
4	62,662.29	0.90	0.131
5	62,083.98	0.85	0.485
6	61,830.01	0.84	0.186

**Table 3:** LQoLP domain scores for the three classes

LQoLP domain	Class 1 (*n* = 358)	Class 2 (*n* = 342)	Class 3 (*n* = 577)	*F* statistic (*df* = 2)	Group differences
Living situation (SD)	4.38 (1.46)	4.45 (1.53)	4.91 (1.3)	16.69*	3 > 2,1
Finances (SD)	3.49 (1.31)	4.3 (1.51)	4.31 (1.31)	46.7*	3,2 > 1
Family relations (SD)	2.93 (1.05)	5.88 (0.75)	5.44 (0.85)	1162.65*	2 > 3 > 1
Safety (SD)	4.68 (1.23)	5.81 (0.71)	5.37 (0.92)	113.44*	2 > 3 > 1
Leisure and social participation (SD)	4.19 (1)	5.33 (0.75)	4.98 (0.85)	160.8*	2 > 3 > 1
Health (SD)	4.07 (0.98)	5.33 (0.77)	4.76 (0.88)	176.44*	2 > 3 > 1
Fulfilment (SD)	4.58 (0.92)	5.83 (0.8)	4.71 (0.73)	264.1*	2 > 3,1
Framework (SD)	5.26 (0.98)	6.34 (0.77)	5.01 (0.76)	284.54*	2 > 1 > 3
Positive esteem (SD)	5.54 (1.16)	6.72 (0.49)	5.02 (0.87)	377.34*	2 > 1 > 3
Negative esteem (SD)	4.01 (1.25)	6.35 (0.85)	4.08 (0.85)	668.29*	2 > 3,1

*p ≤ 0.001

A Chi-square test for equality of distributions revealed no significant differences in how participants from the seven samples were distributed over the three classes *χ*^2^ (12, *N* = 1277) = 10.92, *p* = 0.54.

### Class description

Class 1 (*n* = 358) comprises 28 percent of the sample and encompasses people with severe mental health problems with the lowest score on all of the LQoLP domains, except for two of the intangible domains ‘framework’ and ‘positive esteem’. Individuals in this class reported low scores on the domains ‘family relations’, and ‘leisure and social participation’. Moreover, they score relatively low on the domain ‘health’ despite not receiving more care than the other two classes. Therefore, Class 1 was labelled ‘socially isolated individuals with unmet care needs’.

Involving nearly 27 percent of the sample, Class 2 (*n* = 342) includes people with severe mental health problems with the highest score on every life domain, except on two of the tangible LQoLP domains ‘living situation’ and ‘finances’. Individuals in this class report especially high scores on the domains of the LRI and are therefore labelled ‘individuals with an overall good QoL having a meaning in life’.

Class 3 (*n* = 577; 45.2%) is the largest class and involves people with severe mental health problems who are characterised by the lowest scores on the ‘framework’ and ‘positive esteem’ domains and by the highest scores on the life domains ‘living situation’ and ‘finances’. On the other six domains, individuals in Class 3 report an intermediate score. Since individuals in this class report satisfactory scores on the six tangible domains, but relatively low scores on the domains of the LRI and a high degree of negative affect, Class 3 was labelled individuals with a good overall QoL but lacking a meaning in life and struggling with affect.

### Class comparison

As can be seen in Table 4, there were no significant differences between the classes on most of the sociodemographic variables. No differences were found between the classes for mean age, gender distribution, nationality, and mean age for cessation of formal education. The classes differed on having an intimate relationship, but post hoc tests revealed no significant differences between pairs of classes. The classes also did not differ significantly with regard to having structured daily activities, receiving social benefit, living alone, and marital status.

**Table 4:** Associations between the three latent classes and sociodemographic variables, health-related variables, and measures of well-being

Variable	Class 1 (*n* = 358)	Class 2 (*n* = 342)	Class 3 (*n* = 577)	Statistic^a^ (*p* value)	Group differences
Sociodemographic variables					
Mean age (SD)	35.16 (14.7)	35.18 (15.5)	35.11 (14.6)	*χ* ^2^(H) = 0.05 (0.974)	–
Male	72.8%	74.3%	71.1%	*χ* ^2^ = 1.15 (0.562)	–
Dutch nationality	82.7%	82.2%	84.4%	*χ* ^2^ = 0.85 (0.655)	–
Mean age for cessation of formal education (SD)	15.88 (5.2)	15.52 (6.3)	16.21 (6.7)	*F* = 1.35 (0.259)	–
Intimate relationship	28.4%	35.4%	27.4%	*χ* ^2^ = 0.9.52 (0.049)	–
Structured daily activities	78.5%	77.0%	76.9%	*χ* ^2^ = 0.355 (0.837)	–
Social benefit	62.1%	57.8%	60.3 4	*χ* ^2^ = 1.375 (0.503)	–
Living alone	28.8%	29.8%	30.3%	*χ* ^2^ = 0.258 (0.879)	–
Unmarried	74.4%	76%	76.9%	*χ* ^2^ = 0.737 (0.603)	–
Health-related variables					
Saw a psychiatric care professional during the last year	62%	61.7%	57.2%	*χ* ^2^ = 2.87 (0.238)	–
Hospitalised for psychological complaints during the past year	19%	23.1%	21%	*χ* ^2^ = 1.778 (0.411)	–
Medication for psychological complaints during the last year	59.5%	59.4%	57.4%	*χ* ^2^ = 0.56 (0.757)	–
Admitted to psychiatric hospital/ward	50.7%	55%	53.6%	*χ* ^2^ = 1.372 (0.504)	–
Age at first admission to psychiatric hospital/ward (SD)	25.3 (11.9)	24.8 (12.2)	25.4 (11.4)	*F* = 0.166 (0.847)	–
Unable to gain professional help for health during past year	76 (21.2%)	72 (21.2%)	122 (21.3%)	*χ* ^2^ = 0.00 (0.998)	–
Measures of well-being					
Life satisfaction score (SD)	4.17 (1.24)	4.42 (1.22)	4.33 (1.22)	*F* = 3.74 (0.024)	2 > 1
Cantril’s ladder (SD)	50.67 (23.4)	57.61 (23.1)	54.53 (22.7)	*F* = 7.8 (< 0.001)	2 > 1, 3 > 1
Happiness scale (SD)	2.89 (1)	2.93 (1)	2.95 (1)	*F* = 0.44 (0.643)	–
Negative affect (SD)	4.89 (1.96)	4.53 (1.57)	5.08 (1.65)	*F* = 10.96 (< 0.001)	2 < 1, 2 < 3

^a^Depending on the variable, an ANOVA (*F*), Chi-square test (*χ*^2^), or Kruskall–Wallis test (H) was used

As displayed in Table 4, the classes did not differ significantly on any of the health-related variables. No significant differences were identified for receiving professional help or being hospitalised due to psychological complaints during the past year, nor did the classes differ on taking medication for psychological complaints during the past year, being admitted to a psychiatric ward or hospital, age at first admission, or being unable to gain professional help for their health during the past year.

Table 4 reveals that the classes differed significantly on three of the four measures of well-being. Individuals in Class 2 reported a significantly higher LSS than individuals in Class 1. Moreover, individuals in Class 2 and Class 3 scored significantly higher on Cantril’s Ladder than individuals in Class 1. Additionally, individuals in Class 2 reported significantly less negative effect than individuals in the other two classes. No significant differences were identified for the Happiness Scale.

### Domains contributing to the class differentiation

Table 5 provides the univariate entropy values for the ten LQoLP domains. Univariate entropy values range between 0.041 (domain ‘living situation’) and 0.368 (domain ‘family relations’). The average univariate entropy is 0.177 (SD = 0.112). The domains ‘family relations’ (0.368), ‘positive self-esteem’ (0.366), and ‘negative self-esteem’ (0.231) have the highest univariate entropy values and are most useful for identifying the latent classes.

**Table 5:** Univariate entropy values for the ten LQoLP domains (*N* = 1277)

Quality of life domain	Univariate entropy
Living situation	0.041
Finances	0.056
Family relations	0.368
Safety	0.061
Leisure and social participation	0.131
Health	0.142
Fulfilment	0.180
Framework	0.198
Positive self-esteem	0.231
Negative self-esteem	0.366

## Discussion

Several studies have underlined the heterogeneity and idiosyncratic nature of QoL, warranting a differentiated approach to interpreting and applying QoL data. This study aimed to investigate classes with distinct QoL profiles in a broad group of people with severe mental health problems. To further facilitate the interpretation of QoL scores, the QoL domains which are most strongly related to these classes were examined. Utilising a person-centred method in the form of LCA, three classes with distinct QoL profiles were identified. The results further accentuate the individual nature of QoL, a finding that is in confirmation with previous studies [23, 24].

Closer inspection of the classes based on the ten subjective LQoLP domains, sociodemographic variables, health-related variables, and measures of well-being suggests that QoL is primarily determined by subjective, individual aspects rather than by objective circumstances. Three findings support this notion. First, participants from the seven included studies were divided evenly over the three classes, even though some samples cover (forensic psychiatric) inpatients, while other samples involve outpatients. Differences regarding the QoL of psychiatric inpatients and outpatients have been established in the past [5, 43]. The current results indicate that, even though group averages on the QoL domains may differ between groups, patients from different settings may have similar QoL profiles. Second, the classes differed significantly on a single sociodemographic or health-related variable: ‘having an intimate relationship’. Post hoc tests, however, revealed no differences between pairs of classes on this variable. Many studies report a positive relationship between QoL and several sociodemographic or health-related variables, such as age, being in paid employment, symptoms of depression, and negative schizophrenic symptoms [18, 19, 21, 22, 43]. The lack of differences between the classes on sociodemographic and health-related variables in this study may appear counterintuitive, but many researchers have observed a weak association between objective conditions and an individuals’ subjective appraisal of these conditions [44–46]. This phenomenon is known as the ‘disability paradox’ [47]. The results suggest that a disability paradox is present in the current sample. Third, significant differences were identified for Cantril’s Ladder and the LSS, which reflect participants’ subjective evaluations of their objective circumstances. Moreover, individuals in Class 2 reported significantly lower negative affect than the other classes, which is likely to contribute to their high scores on the ten LQoLP domains. This explanation sits well with studies in which an association between affect and subjective QoL has been identified [48, 49].

The notion that QoL is primarily determined by subjective, individual aspects rather than by objective circumstances is in agreement with the theory of Subjective well-being homeostasis [50, 51]. According to the theory of SWB homeostasis, an individuals’ SWB is homeostatically regulated to vary within a relatively narrow range of genetically determined set-points [50, 52]. According to this theory, objective circumstances do influence SWB, but only within a genetically determined bandwidth. It is possible that the QoL profiles identified in this study reflect different set-points rather than objective circumstances. Bartels [53] provided additional evidence for the genetic component of QoL and SWB. In a review of 30 twin studies on the genetic component of well-being, heritability estimates ranging from 17 to 56 percent for overall well-being, and 22–42 percent for QoL were identified.

To facilitate the interpretation of QoL scores, the LQoLP domains that were most strongly related to the classification were identified. Based on univariate entropy scores, the domains ‘family relations’ and ‘self-esteem’ were most useful for identifying the latent classes. This means that the classes are most clearly demarcated on these domains [38]. Individuals in Class 1 score exceptionally low on family relations (2.93), well below the cut-off score of 4 [5]. In contrast, Class 2 and 3 score very high on this domain. The large differences between the classes may be explained through the degree of support individuals receive from their family network, which has been found to influence the way patients evaluate their family situation [54]. Additionally, lack of support from family is related to internalised stigma [55]. Scores on Self-esteem (both positive and negative) also differ strongly between the classes. Individuals in Class 2 report significantly higher self-esteem than individuals in the other two classes. The polarising role of self-esteem may be related to stigmatisation, which is known to have a negative impact on self-esteem in people with severe mental health problems [16, 56].

The association between socioeconomic conditions and mental health and QoL is well documented [57–60]. The three profiles identified in this study, however, showed a marked difference in QoL, but not on sociodemographic characteristics. It is possible that the three profiles are indicative of a difference in resilience. Individuals in Class 2 may be better equipped to endure adversities caused by their poor mental health and socially adverse positions, while individuals in Class 1 and 3 are not as equipped to do so. The results suggest that the ability to discern meaning and purpose in one’s life may be important in explaining this difference in resilience. Studies by Min and colleagues [61] and Wartelsteiner and colleagues [62] confirm this notion.

### Strengths and limitations

The current study was based on a large database of LQoLP data. The comprehensiveness of the LQoLP and the rigidity of its development ensure data of high quality. The use of a person-centred method in the form of LCA enabled us to better capture the multidimensional nature of QoL. Apart from these strengths, three weaknesses should be kept in mind when interpreting the results. First, the analysis was limited to LQoLP data. These domains are based on thorough empirical research [25, 26], but as most QoL scales tend to assess slightly different QoL domains, it is possible that classes with different profiles would have been found if another QoL measure had been used. The second limitation relates to the timespan in which data were collected. Data were collected in the period between 1997 and 2012, a span of 15 years. Changes in society and in mental healthcare [63, 64] may have influenced the meaning and composition of QoL for people with psychiatric problems, which might have biased the results. Third, no clinical data were available for the characterisation of the classes. Past research indicates that variables such as type and severity of symptoms, style of coping, and adherence to treatment are related to QoL [20, 21, 46]. This type of data would have provided additional insight into the nature of the three classes, and future studies may include them.

## Conclusion

The identification of three classes with distinct QoL profiles for people with severe mental health problems further emphasises the heterogenic nature of QoL in this population. The classes differed markedly on the subjective QoL domains, general well-being, and negative affect, but not on the majority of the sociodemographic variables and objective indicators of QoL. This result suggests that, for people with severe mental health problems, QoL is primarily determined by individual, personal aspects rather than circumstances, and provides additional evidence for the disability paradox. Furthermore, the results stress the importance of subjective evaluations in the assessment of the QoL of people with severe mental health problems. The QoL profiles may aid in the interpretation of QoL scores and the domains ‘family relations’ and the two domains related to self-esteem are especially useful in this regard.
